# The Overlooked Diagnosis: A Case Report on Autonomic Dysfunction and Chronic Dizziness in an Elderly Patient With Neuroimaging Correlation

**DOI:** 10.7759/cureus.86590

**Published:** 2025-06-23

**Authors:** Saran AK, Tribhuwan Kumar, Md. Zabihullah, Gagan K Banodhe, Raihan Mannan

**Affiliations:** 1 Physiology, All India Institute of Medical Sciences, Patna, Patna, IND

**Keywords:** autonomic dysfunction, chronic dizziness, elderly, heart rate variability, small vessel ischemia, tilt table test

## Abstract

Dizziness is a common yet diagnostically challenging complaint in the elderly, often resulting from multifactorial causes such as vestibular dysfunction, autonomic impairment, and cerebrovascular disease. This case report describes a 74-year-old woman with persistent, non-vertiginous dizziness, exacerbated by postural changes, but without orthostatic hypotension or classic vestibular signs. Comprehensive evaluation, including autonomic function tests, tilt-table testing, and neuroimaging, revealed autonomic dysfunction and chronic small-vessel ischemic changes in the periventricular white matter, along with a small hypodense lesion in the pons. Specifically, the patient exhibited reduced heart rate variability, impaired parasympathetic reflexes, and cerebrovascular alterations suggestive of disrupted autonomic pathways. This case underscores the importance of integrating autonomic testing with neuroimaging to elucidate the underlying pathophysiology of chronic dizziness in elderly patients.

## Introduction

Dizziness is a common and disabling symptom in older adults, affecting approximately 30% of individuals over 60 years of age globally [1] and nearly 15% of Indian adults aged 65 and older [2]. It often presents as a chronic complaint and is associated with a decline in health-related quality of life, impaired postural control, and functional limitations in daily life [3,4]. Although vestibular dysfunction is a primary and well-recognized cause, emerging evidence highlights the role of autonomic dysregulation and cerebral small vessel disease (CSVD) as significant but frequently overlooked contributors to unexplained dizziness in this population.

Autonomic dysfunction is a well-documented cause of non-vertiginous dizziness, typically presenting as intermittent episodes of lightheadedness or mental fogginess triggered by orthostatic stressors, such as rising from a supine position or prolonged standing [5]. However, the pathophysiology of chronic, persistent dizziness remains poorly understood, and systematic evaluation using standardized autonomic function testing is rarely performed. Notably, in a study that systematically excluded other known causes, approximately 80% of patients with persistent dizziness of unknown origin were found to have at least one autonomic abnormality, pointing to a substantial diagnostic gap [6].

Dysautonomia may result from diverse etiologies, including primary autonomic failure, diabetes, autoimmune disorders, chronic infections, and age-related neurodegeneration [7]. In addition, cerebral small vessel ischemia may disrupt autonomic regulatory circuits in the brainstem, contributing to subclinical or unexplained autonomic symptoms [8]. A comparative neuroimaging study found that patients with dizziness who lacked a vestibular diagnosis exhibited a significantly greater burden of white matter abnormalities, suggestive of CSVD and implicating a central mechanism in “unexplained” cases [9].

These overlooked contributors can be systematically evaluated through neuroimaging modalities such as magnetic resonance imaging (MRI) and computed tomography (CT), along with autonomic function tests (AFTs) like tilt-table testing and heart rate variability analysis, which together allow detailed assessment of autonomic regulation and potential cerebrovascular contributions.

To date, only a few studies have investigated the combined diagnostic utility of autonomic testing and neuroimaging in patients with chronic dizziness. This case report presents an integrated approach employing both modalities and aims to underscore the diagnostic importance of identifying underrecognized autonomic and cerebrovascular contributors in older adults with chronic, otherwise unexplained dizziness.

## Case presentation

Clinical history

A 74-year-old female presented with a three-month history of non-vertiginous dizziness, described as a persistent background sensation of lightheadedness and imbalance, with superimposed intermittent exacerbations lasting approximately 10-15 minutes. These transient worsening episodes occurred multiple times daily, particularly triggered by standing or sudden postural changes. There was no associated loss of consciousness, visual disturbances, palpitations, tinnitus, or hearing loss.

Additional complaints included generalized weakness, occasional muscle cramps, and a sensation of heaviness in the left leg. She also reported disturbances in sweating, specifically focal hyperhidrosis involving the hands and feet.

The patient had a history of well-controlled essential hypertension for the past three years, managed with amlodipine 10 mg once daily. She had no history of coronary artery disease, myocardial infarction, heart failure, arrhythmias, or peripheral arterial disease, and denied symptoms suggestive of cardiovascular or peripheral vascular insufficiency, such as exertional angina, orthopnea, paroxysmal nocturnal dyspnea, palpitations, or intermittent claudication. A former smoker with a cumulative exposure of approximately 2.5 pack-years, she had quit five years prior. There was no personal or family history of diabetes mellitus, cerebrovascular accidents, neurodegenerative disorders, or vestibular dysfunction.

Examination and initial workup

On examination, the patient’s blood pressure was 132/78 mmHg in the sitting position, with a resting heart rate of 77 beats per minute (bpm). Her body mass index (BMI) was 24.2 kg/m². Neurological examination revealed mild lower limb weakness (power 4/5), but cranial nerves, reflexes, and cerebellar signs were normal. Gait assessment showed mild unsteadiness, particularly with eyes closed.

The Dix-Hallpike maneuver, a diagnostic test for benign paroxysmal positional vertigo, was performed to assess for a vestibular cause of dizziness and yielded negative results. Routine laboratory investigations, including thyroid function tests, serum vitamin B12, glycated hemoglobin (HbA1c), and renal function parameters, were all within normal limits. 

Investigations

AFTs were performed to evaluate cardiovascular autonomic regulation. Short-term (five-minute) heart rate variability analysis demonstrated time domain values near the lower limit of normal and reductions in frequency domain parameters, indicating autonomic dysfunction (Table 1). Specifically, the standard deviation of normal-to-normal intervals (SDNN) was 19.27 milliseconds (ms), and the root mean square of successive differences (RMSSDs) was 21.26 ms. In the frequency domain, total power was reduced at 439.1 ms². Low-frequency (LF) power measured 65.2 ms² (24.91 normalized units), while high-frequency (HF) power was 193.3 ms² (73.83 normalized units). The LF/HF ratio was markedly low at 0.34, indicating altered sympathovagal balance with a relative predominance of parasympathetic modulation.

**Table 1:** Short-term (five-minute) heart rate variability parameters ms: milliseconds; ms^_2_^: milliseconds squared; SD: standard deviation

Parameter	Observed value	Reference range (mean ± SD)
Time domain parameters		
Average R-R Interval (ms)	834.5	926 ± 90
Standard deviation of all normal to normal R-R Intervals (SDNN) (ms)	19.27	50 ± 16
Root mean square of successive differences (RMSSD) (ms)	21.26	42 ± 15
Frequency domain parameters		
Low-frequency power (LF, 0.04–0.15 Hz) (ms²)	65.2	1170 ± 416
LF power (normalized units)	24.91	54 ± 4
High-frequency power (HF, 0.15–0.45 Hz) (ms²)	193.3	975 ± 203
HF power (normalized units)	73.83	29 ± 3
Total power (sum of all spectral components) (ms²)	439.1	3466 ± 1018
Low-frequency to high-frequency ratio (LF/HF ratio)	0.3373	1.5 - 2.0

As part of Ewing’s battery of cardiovascular autonomic reflex tests, the Valsalva ratio was 1.93 (normal: >1.21), within normal limits. The 30:15 ratio from the lying-to-standing test was 1.07 (normal: >1.03), also within the normal range. By contrast, the deep breathing test revealed a delta heart rate of 3.94 bpm (normal: ≥10 bpm), suggestive of impaired cardiovagal modulation. The isometric handgrip test showed a blunted diastolic blood pressure increase of 4 mmHg (normal: >15 mmHg), indicating impaired sympathetic reactivity.

The tilt-table test, a supplementary assessment, revealed no evidence of orthostatic hypotension (defined as a sustained fall in systolic blood pressure (SBP) ≥ 20 mmHg or diastolic blood pressure (DBP) ≥ 10 mmHg within three minutes of tilt) and no delayed orthostatic drop (beyond three minutes). A modest heart rate increase of 8 bpm at three minutes, along with stable blood pressure, indicated generally preserved baroreflex-mediated sympathetic activation (Table 2).

**Table 2:** Hemodynamic response to tilt-table testing SBP: systolic blood pressure; DBP: diastolic blood pressure; HR: heart rate; mmHg: millimeters of mercury; bpm: beats per minute; min: minute

Time (minutes)	SBP (mmHg)	DBP (mmHg)	HR (bpm)
Basal	117	69	70
Immediately after tilt	128	71	73
1 min	130	71	76
3 min	132	70	78
5 min	134	71	78
7 min	137	71	76
10 min	133	71	77
13 min	131	70	78
15 min	127	68	77
18 min	119	69	79
20 min	116	69	80
Immediately after tilt back	123	76	76
1 min	123	68	72
2 min	120	68	73
5 min	120	69	73

**Figure 1:**
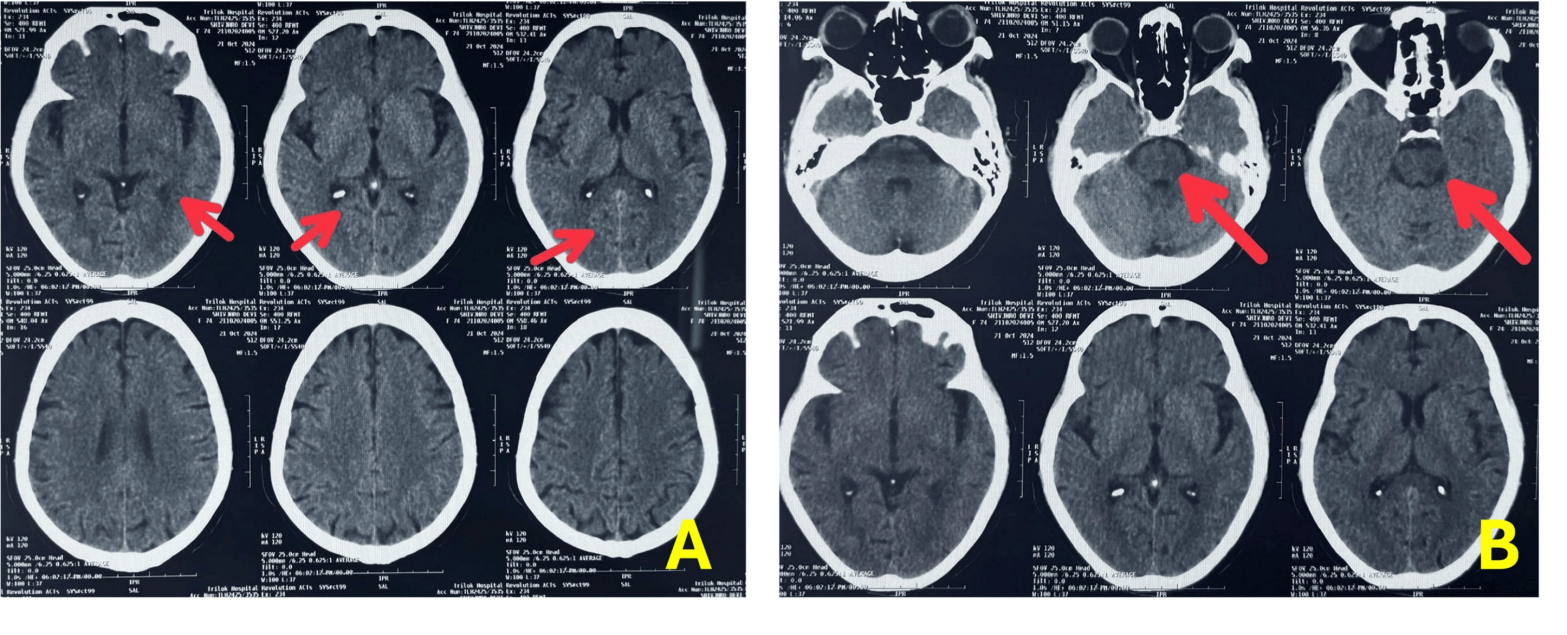
(A) Axial non-contrast computed tomography (NCCT) of the brain at the level of the lateral ventricles showing bilateral periventricular white matter hypodensities (red arrows), consistent with chronic small-vessel ischemic changes. (B) Axial NCCT at the pontine level demonstrating a hypodense lesion (HU 7-11) in the left pons (red arrows).

Neuroimaging with non-contrast computed tomography (NCCT) revealed a small hypodense lesion in the left pons (HU 7-11), along with bilateral periventricular white matter hypodensities, findings consistent with chronic small vessel ischemic changes characteristic of CSVD (Figure 1A, 1B).

The patient was initially managed empirically by the treating team with vestibular suppressants, considering the chronic nature of her dizziness and the absence of a definitive diagnosis. Following the identification of autonomic dysfunction in the context of chronic small vessel ischemic changes on neuroimaging, a more targeted, pathophysiology-based management strategy was instituted.

Pharmacological management initially included cilnidipine 10 mg once daily, which was later modified to a fixed-dose combination of telmisartan and amlodipine for optimal blood pressure control. In addition, aspirin 150 mg once daily was prescribed as secondary prophylaxis against cerebrovascular events. Non-pharmacological measures were directed at enhancing autonomic stability and mitigating fall risk. These included advice on maintaining adequate oral hydration, patient education on gradual postural transitions, maintenance of regular sleep-wake patterns, and home safety modifications. On subsequent follow-up, the patient reported persistent but less frequent exacerbations of dizziness, suggesting partial symptomatic improvement. Continued monitoring was advised to assess the progression of autonomic dysfunction and to address any evolving neurological or vascular concerns.

## Discussion

Chronic dizziness in elderly patients often presents a complex diagnostic challenge due to its multifactorial etiology, with contributions from vestibular, cardiovascular, neurological, and autonomic systems [10]. This case illustrates such complexity in a 74-year-old female, where reduced short-term heart rate variability, impaired parasympathetic reactivity on deep breathing, and blunted sympathetic reactivity on isometric handgrip pointed toward autonomic dysfunction. Neuroimaging findings of cerebral small vessel disease with a focal pontine lesion further supported central autonomic involvement. The negative Dix-Hallpike maneuver reduced the likelihood of a vestibular origin.

The patient’s resting autonomic tone, assessed through short-term heart rate variability, showed evidence of autonomic dysregulation, with an SDNN of 19.27 ms, RMSSD of 21.26 ms, total power of 439.1 ms², LF power of 65.2 ms², HF power of 193.3 ms², and an LF/HF ratio of 0.34. Even after accounting for age-related normative variation, the observed reduction in these parameters is indicative of impaired autonomic modulation and has been associated with increased cardiovascular and all-cause mortality in the elderly [11]. Parasympathetic function was notably impaired, as evidenced by the deep breathing test. The average delta heart rate, 3.94 bpm, fell well below the normal threshold of ≥10 bpm for elderly individuals, reflecting reduced cardiovagal tone. However, other parasympathetic reactivity indices, including the Valsalva ratio (1.93) and the 30:15 ratio (1.07), were within normal limits. This suggests a selective or partial impairment in parasympathetic pathways, particularly affecting respiratory sinus arrhythmia, a known early marker of parasympathetic dysfunction [12,13].

Baroreceptor-mediated sympathetic responses were preserved, as shown by the tilt-table test. There was no orthostatic hypotension or excessive postural tachycardia, with a modest rise of 8 bpm in heart rate at the third minute after tilting. This suggests intact baroreflex integrity. By contrast, non-baroreceptor-mediated sympathetic vasomotor reactivity, measured by the isometric handgrip test, was blunted, with only a 4 mmHg rise in diastolic blood pressure. This pattern, preserved baroreflex but impaired vasomotor sympathetic activity, is consistent with age-related or central autonomic dysfunction [14].

Neuroimaging added valuable insight, revealing a small hypodense lesion (HU 7-11) in the left pons and periventricular white matter changes typical of CSVD. These findings are significant, as the pons harbours key structures of the central autonomic network (CAN), a highly integrated system comprising the insular cortex, hypothalamus, amygdala, periaqueductal gray, parabrachial nucleus, and medullary centers like the nucleus tractus solitarius, which coordinate afferent and efferent autonomic signals [15]. CSVD, through chronic hypoperfusion and white matter disconnection, has been shown to compromise CAN integrity, leading to dysautonomia [16]. Moreover, Ibitoye RT et al. have shown a link between frontal white matter integrity and dizziness in patients with cerebral small vessel disease, further supporting the relevance of neurovascular disruption in symptom generation [17]. Importantly, autonomic dysfunction may not merely be a consequence but also a contributor to neurodegeneration, as reduced vagal tone is associated with systemic inflammation and decreased cerebral perfusion, thereby perpetuating cognitive decline and functional impairment [18]. This bidirectional relationship warrants early recognition and a multidisciplinary approach.

This report has a few limitations. Autonomic function tests may be influenced by physiological and external factors, which can affect consistency across sessions. Continuous electrocardiogram or blood pressure monitoring was not performed, which might have provided additional insights into subtle or intermittent autonomic changes. In addition, sudomotor testing, such as the quantitative sudomotor axon reflex test (QSART) or thermoregulatory sweat testing (TST), was not conducted, limiting the assessment of sympathetic cholinergic function. As this is a single-case observation with a cross-sectional assessment, causal inferences between CSVD and autonomic dysfunction should be made cautiously.

## Conclusions

This case illustrates the subtle yet clinically meaningful role of central autonomic dysfunction in an elderly patient with chronic dizziness and CSVD. The patient exhibited reduced heart rate variability, impaired parasympathetic function, and diminished non-baroreceptor sympathetic responses, despite normal findings on tilt-table and vestibular assessments. Neuroimaging revealed periventricular white matter changes and a small hypodense lesion in the pons (HU 7-11), suggesting disruption of central autonomic regulatory pathways. These findings highlight the diagnostic value of integrating autonomic function testing with neuroimaging, particularly when routine cardiovascular and vestibular evaluations are inconclusive. Clinicians should maintain a high index of suspicion for dysautonomia in elderly patients with unexplained dizziness. This case adds to the growing recognition of the interplay between CSVD and autonomic impairment and underscores the need for multidisciplinary assessment and long-term follow-up. Such an approach may improve diagnostic precision and support more nuanced and individualized management strategies, including non-pharmacological interventions, in similar clinical scenarios.
